# Energy flows with intentional changes in leg movements during baseball pitching

**DOI:** 10.3389/fspor.2025.1534596

**Published:** 2025-04-08

**Authors:** Ryota Matsuda, Yuichi Hirano, Jun Umakoshi, Arata Kimura

**Affiliations:** ^1^ Graduate School of Sports and Health Studies, Hosei University, Machida-shi, Japan; ^2^ Department of Sports and Health, Faculty of Sports and Health Studies, Hosei University, Machida-shi, Japan

**Keywords:** biomechanics, baseball pitching, energy flow, lower extremity, trunk

## Abstract

Baseball pitchers are typically required to generate high ball velocity in their pitches. Many studies have focused on the lower extremity movements engaged at the beginning of the pitching motion to generate high ball velocity. It is assumed that the change in movement of the lower extremity induces the change in energy flow in pitching because the lower extremity generates high mechanical energy transferred to the ball. However, no studies have focused on the effects of intentional changes in lower extremity movements on energy flow. This study examined how altering stride length changes the energy flow from the lower extremities to the trunk. Twenty male college baseball pitchers participated in this study. In addition to pitching with normal stride length (NS), they pitched with under-stride length (US) and over-stride length (OS), defined as ±20% of NS. The positive and negative work of joint power, the sum of joint force power and segment torque power, were analyzed at the pivot hip, stride hip, and trunk joint. Positive work was defined as energy inflow to the lower torso from each joint, while negative work was defined as energy outflow from the lower torso to each joint. These values were then compared across stride length conditions. Our results showed that the energy inflow from the pivot hip to the lower torso and outflow from the lower torso to the stride hip changed with stride length during each phase. However, the total energy outflow from the lower torso to the trunk joint during the stride and arm-cocking phase was not significantly different with stride length (*p* = 0.59; *η*^2^ = 0.02), and the ball velocity did not significantly differ between the US and OS (*p* = 1.00; *d* < 0.01). This study highlights that altering stride length might not lead to changes in total energy outflow from the lower torso to the trunk joint, implying difficulties in explaining ball velocity only by the lower extremity mechanics.

## 1 Introduction

Baseball pitchers are typically required to generate high ball velocity in their pitches. Many researchers have studied pitching to improve ball velocity (1–3). The pitching consists of a sequence of full-body movements that begin when the pitcher lifts the stride leg, progresses with a translation toward the home plate, and culminates in the throw after landing the stride foot and rotating the trunk. A lot of the studies have focused on the lower extremity movements engaged at the beginning of the pitching motion for generating high ball velocity (4–8).

Stride length is a crucial parameter of the lower extremity mechanics for generating high ball velocity. From a theoretical standpoint, the extended stride length allows for an increase in total body linear momentum in the throwing direction, which ultimately transfers to the ball. It has been reported that the stride length is one of the predictive parameters of ball velocity, and there was a significant correlation between the stride length and ball velocity in mature baseball pitchers (9–12). The results of the cross-sectional studies were consistent with the theoretical standpoint, while some previous studies altering stride length within the individuals did not show it. It has been reported that there was no significant difference in ball velocity between the over-stride length and under-stride length conditions (13). This previous study also reported that peak propulsion ground reaction force (GRF) was statistically greater for the pivot leg with extending stride length, and breaking GRF was statistically greater for the stride leg with extending stride length. It remains unclear why there is no significant difference in ball velocity despite changes in stride length and GRF.

Energy flow analysis is one way to address this issue. It allows us to quantify the generation and absorption of energy at the joints and the transfer of energy between segments in human movement. Baseball pitching is recognized as a kinetic chain in which the mechanical linkages of body segments allow for the sequential transfer of forces and energy (14–16). Researchers have used energy flow analysis to investigate how energy is generated, absorbed, and transferred through the kinetic chain (17–20). Specifically, the lower extremity generates a large amount of energy that is transferred to the trunk and then transferred to the upper extremities, ultimately contributing to the ball release (18–20). Then, it is necessary for the lower extremities to generate more energy and transfer it to the trunk without loss for high ball velocity. From the perspective of energy analysis, it is assumed that the energy generated in the lower extremity would be greater with the intentionally extending stride length compared to the shortening stride length. If the energy generated in the lower extremity were transferred to the hand without any loss, the ball velocity would be greater with the extending stride length than the shortening stride length. However, previous studies showed no significant difference in ball velocity between the over-stride and under-stride lengths (13, 21). The results imply that there is a loss in energy flow somewhere from the lower extremity to the hand. Therefore, energy flow analysis is crucial to address the issue of why there is no significant difference in ball velocity despite changes in energy generation by examining not only the energy generation at the lower extremity but also the energy exchange from the lower extremity to the trunk.

Previous pitching research on energy flow has primarily focused on how energy flow relates to ball velocity or upper extremity joint loading (17, 22). However, no studies have focused on the effects of intentional changes in lower extremity movements on energy flow. Investigating energy flow in altered stride length pitching would enhance understanding why ball velocity does not differ statistically between the over-stride and under-stride pitching and provide useful information to pitch high ball velocity. Therefore, this study examined how altering stride length affects the energy flow from the lower extremity to the trunk in baseball pitching.

## 2 Methods

### 2.1 Participants

The sample size for this study was calculated using a two-way ANOVA with a power of 80%, an alpha error of 0.05, and an effect size of 0.3, which indicated that sixteen participants were required. To ensure robustness, twenty male college baseball pitchers (age: 19.9 ± 1.1 years; height: 173.2 ± 5.8 cm; mass: 71.8 ± 6.4 kg) participated in this study. The exclusion criteria were sidearm and underarm throwers, injured individuals, and those unable to throw maximal effort. We verbally assessed the participants' injury histories using the following criteria: (1) no surgical procedures within the past year, (2) no current pain in any part of the body, and (3) the participant can throw with maximal effort. All participants provided written informed consent to participate in this study, which was conducted in accordance with the Declaration of Helsinki and approved by the Ethical Committee of the Graduate School of Sports and Health Studies at Hosei University (2022_26).

### 2.2 Stride length determination and experimental protocol of pitching in each stride length

**Figure 1:**
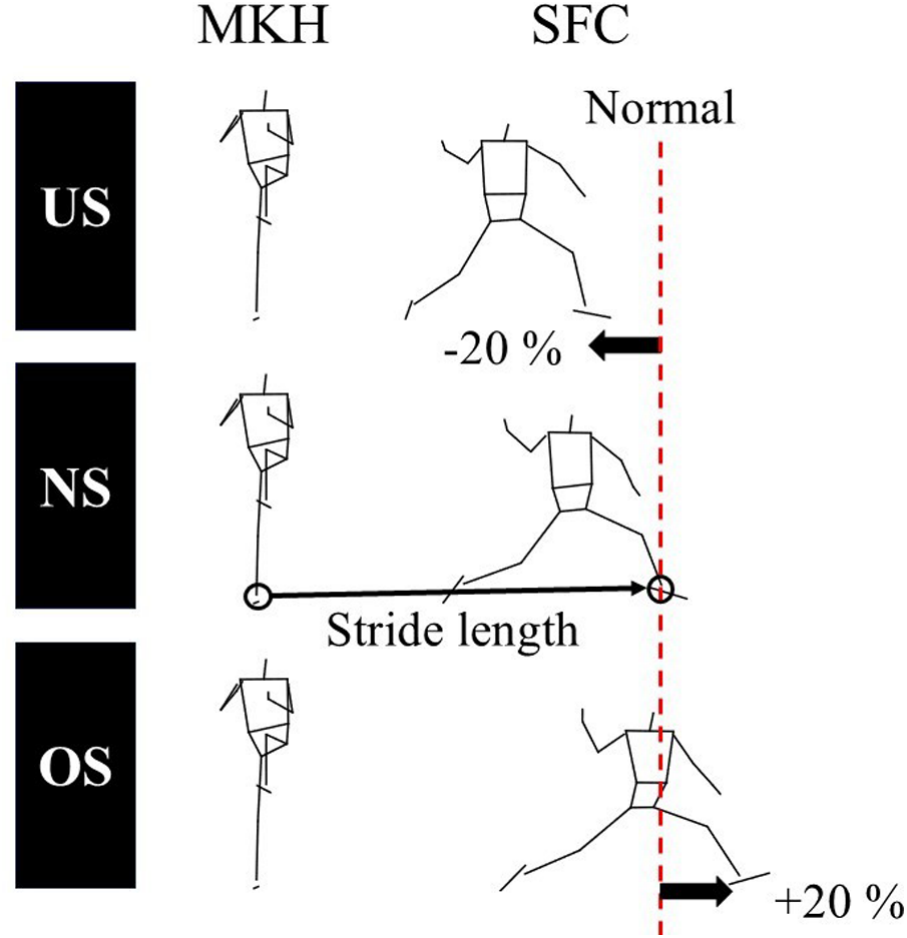
The definition of the stride length. The stride length was defined as the distance from the center of the ankle joint of the pivot leg at the maximal stride knee height (MKH) to that of the stride leg at the stride foot contact (SFC). The under-stride length (US) and over-stride length (OS) were defined as ±20% length of the normal stride length (NS), respectively.

The participants pitched on two indoor flat force platforms (Custom FP, Bertec Inc., Ohio, USA). A set of 47 reflective markers was attached to the skin over specific anatomical landmarks according to a previous study (17), and three reflective markers were attached to the index fingertip, the middle fingertip, and the interphalangeal joint of the middle finger. A set of 4 reflective markers were attached to the ball. After completing a comfortable warm-up, the participants pitched five fastballs with a maximal effort to a target zone 16 m away to determine the normal stride length (NS), following confirmation that they could throw with maximal effort. The three trials with the fastest recorded ball velocities were selected to determine the normal stride length. The stride length was defined as the distance from the center of the ankle joint of the pivot leg at the maximal stride knee height to that of the stride leg at the stride foot contact (Figure 1). The mean stride length within three trials was defined as the NS. Previous studies defined the under-stride length (US) and over-stride length (OS) as the ±25% length of the NS (13). However, the US and OS in this study were defined as the ±20% length of the NS (Figure 1). This is because many participants failed to reach the OS length in the preliminary experiments when the OS was set at +25% of the NS as in previous studies.

To pitch with the same stride length, points on the force plate were marked to indicate stride foot placement for individual stride length conditions (US, NS, or OS). We ensured participants had unlimited time to familiarize themselves with each stride length condition before proceeding with the trials. After practicing pitching with each stride length condition, the participants pitched fastballs until ten balls reached the target zone. The order of conditions was randomized, with measurements for each condition conducted at 1-week intervals. Stride length conditions were randomized using a random number table to minimize the effects of learning or fatigue.

### 2.3 Data collection and analysis

A motion capture system consisting of 13 cameras (VICON MX, Vicon Motion Systems, Ltd., Oxford, UK) was used to record the motion of the reflective markers at a sampling rate of 250 Hz. The cameras were positioned to ensure varied camera heights and angles to capture movements from multiple perspectives. The calibration was conducted around the two force plates performing the pitching movement to ensure accurate motion capture. A standard wand-based calibration protocol spatially aligned the camera system and ensured measurement accuracy across the capture volume. The GRF was recorded using two force platforms at a sampling rate of 1,000 Hz, and the values were synchronized with the motion data. The global coordination system (GCS) was the right-handed orthogonal coordinate system defined as follows: the *Z*-axis was defined as the vertical upward direction; the *Y*-axis was defined as the throwing direction; the *X*-axis was defined as the cross product of the *Y*-axis and *Z*-axis.

**Figure 2:**
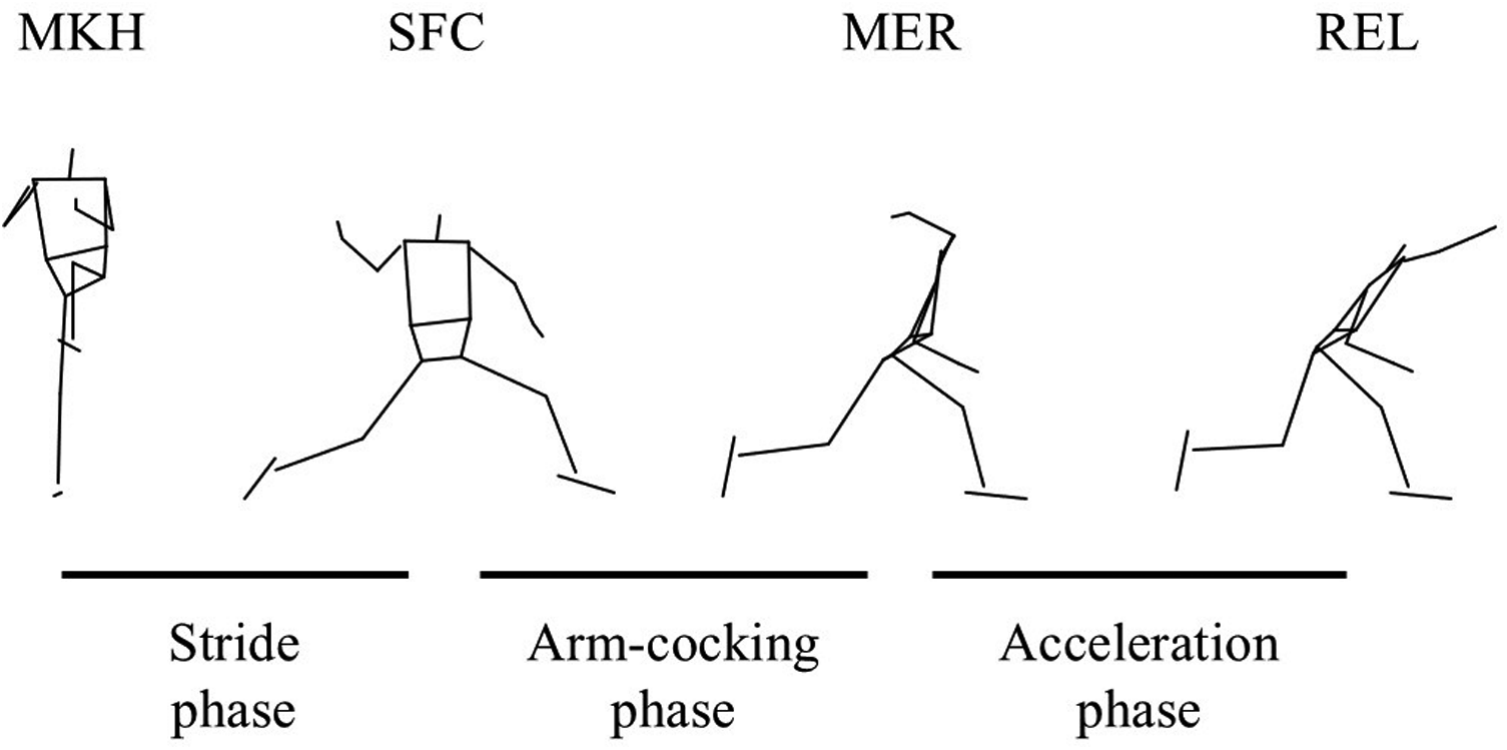
The phase and events in pitching. The stride phase was defined from the maximal knee height (MKH) to the stride foot contact (SFC). The arm-cocking phase was defined from the SFC to the maximal external rotation (MER) of the shoulder. The arm-acceleration phase was defined from the MER to the ball release (REL). These definitions conformed to the previous studies (23).

The pitching motion was divided into three phases, as previously defined (23) (Figure 2). The stride phase was from the maximal knee height (MKH) of the stride leg to the stride foot contact (SFC). The arm cocking phase was from SFC to maximal external rotation (MER) of the throwing arm. The arm acceleration phase was from MER to ball release (REL). The SFC was defined as the instant when the vertical GRF of the stride foot exceeded 5% of body mass (13). The MER was identified as the local maximum of humeral long-axis rotation relative to the thorax between SFC and REL and determined using a *Z*-*Y*'-*Z*' Euler rotation sequence. The REL was defined as the instant when the distance between the center of the ball and the marker on the index fingertip increased by more than 2 cm (24).

The position of the markers and the GRF data were smoothed by applying a fourth-order Butterworth low-pass filter. The cut-off frequency for each marker between 2 and 20.3 Hz was calculated by the residual analysis (25), and for the GRF data was 15 Hz to prevent artifacts from appearing in the joint moment (26, 27). In addition, the GRF data were downsampled from 1,000 to 250 Hz using a cubic spline to synchronize with the motion data.

The whole body was modeled as 16 rigid link segments. Each segment and joint coordination system was defined according to a previous study (17). The inertial parameters for each segment were estimated according to the definition of a previous study (28).

Each segment angular velocities were calculated as:

$$\omega_{x}\;=\;k\;\cdot \;\frac{dj}{dt},\;\omega_{y}\;=\;i\;\cdot \;\frac{dk}{dt},\;\omega_{z}\;=\;j\;\cdot \;\frac{di}{dt}\;$$

where i,j,andk are the unit vectors of each axis of each segment coordination system in Local Coordinate System (LCS). After that, each segment angular velocities were transformed to the magnitude in GCS from that in LCS.

The mechanical energy of the lower torso was calculated as:

$$E\;=\;mgh\;+\;\frac{1}{2}mv^{2}\;+\;\frac{1}{2}(I_{x}{\omega_{x}}^{2}\;+\;I_{y}{\omega_{y}}^{2}\;+\;I_{z}{\omega_{z}}^{2})$$

where mgh is the potential energy, $\frac{1}{2}mv^{2}$ is the translational kinetic energy, $\frac{1}{2}(I_{x}{\omega_{x}}^{2}\;+\;I_{y}{\omega_{y}}^{2}\;+\;I_{z}{\omega_{z}}^{2})$ is the rotational kinetic energy, *m* is the mass of the lower torso, ***g*** is the acceleration of gravity, *h* is the height of the center of mass (COM) of the lower torso, ***v*** is the velocity of the COM of the lower torso, $I_{x},\;\;I_{y},\;\mathrm{and}\;I_{z}$ are the moments of inertia of each axis, $\omega_{x},\;\omega_{y},\;\mathrm{and}\;\omega_{z}$ are the angular velocities of each axis in GCS. The mechanical energy of the lower torso was normalized by body mass.

The joint force power (JFP) was calculated as:

JFP=f⋅v

where ***f*** is the joint force, ***v*** is the joint velocity.

The segment torque power (STP) was calculated as:

STP=τ⋅ω

where τ is the joint torque and ω is the segment angular velocity, respectively. The JFP and STP were normalized by body mass.

The joint power (*JP*) was calculated as:

JP=JFP+STP

**Figure 3:**
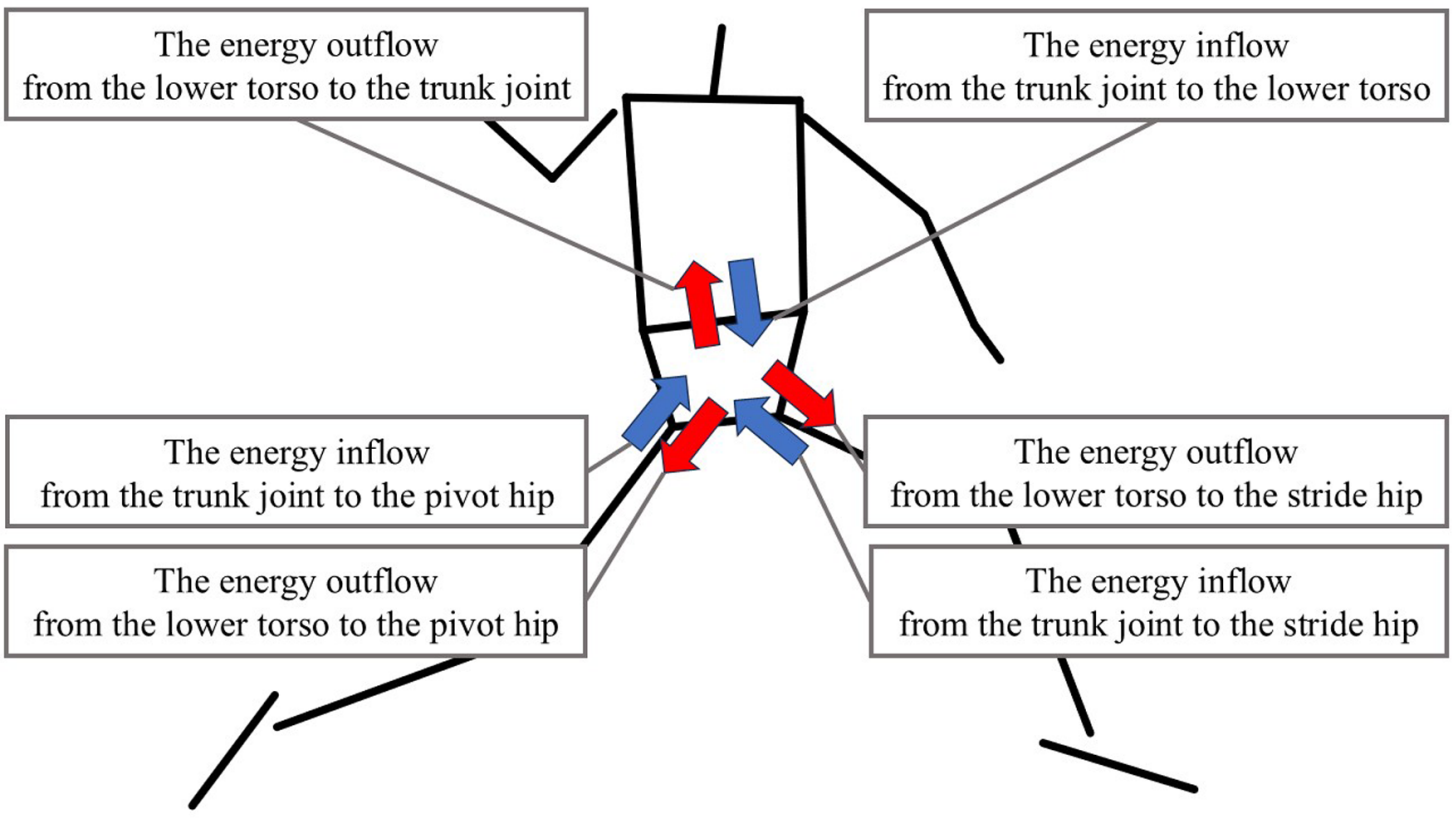
The definition of the energy inflow and outflow. The blue arrow shows the energy inflow from each joint to the lower torso. The red arrow shows the energy outflow from the lower torso to each joint.

The work of *JP* at the pivot hip, stride hip, and trunk joint was calculated by integrating *JP* per phase. The positive value in the work indicates the energy inflow from each joint to the lower torso, and the negative value means the energy outflow from the lower torso to each joint (Figure 3).

The segment power (*SP*) of the lower torso was calculated as:

$$\;SP=\;\overset{n}{\underset{i\;=\;1}{\sum }}\;JP\;$$

where ***i*** is the number of adjacent joints to the segment. The work of *SP* of the lower torso was calculated by integrating the *SP* of the lower torso per phase. The positive value in the work indicates the total energy inflow to the lower torso, and the negative value means the total energy outflow from the lower torso. All data analyses were performed using Python (3.11.7 for Windows).

### 2.4 Statistical analysis

The means and standard deviations (SD) were calculated for all variables. The energy flow to the lower torso between MER and REL has a negligible effect on the magnitude of the ball velocity. Therefore, the three events, MKH, SFC, and MER, in the mechanical energy of the lower torso were included in the statistical analysis, not REL. The two phases, the stride phase and the arm-cocking phase, in each work were included in statistical analysis, not included in the acceleration phase.

Two-way analysis of variance (ANOVA) [conditions (US, DS, OS) × events (MKH, SFC, MER) or phases (the stride phase, the arm-cocking phase)] was conducted to investigate the relationship between the stride length and mechanical energy and each work. One-way ANOVA was conducted to investigate the relationship between the stride length and the ball velocity. For all ANOVA tests, partial eta squared (*η*^2^) was calculated to assess the effect size for main effects and interactions, where values of 0.01, 0.06, and 0.14 indicate small, medium, and large effects, respectively. Tukey honest significant difference (HSD) was conducted for a follow-up analysis in the case of significant main effects and interactions. Cohen's *d* was calculated for *post hoc* pairwise comparisons using Tukey HSD, where values of 0.2, 0.5, and 0.8 indicate small, medium, and large effects, respectively. The level of significance for all comparisons was *p* < 0.05. All statistical analyses were performed using Python (3.11.7 for Windows).

## 3 Results

The mean stride lengths during pitching with the US, NS, and OS were 1.08 ± 0.13 m, 1.35 ± 0.12 m and 1.56 ± 0.13 m (62.52 ± 6.99%, 78.20 ± 5.70% and 89.86 ± 6.58% normalized by body height), respectively. The mean ball velocities with the US, NS, and OS were 32.48 ± 1.72 m/s, 33.90 ± 1.86 m/s and 32.48 ± 1.70 m/s, respectively. There was a significant difference in the ball velocity for the stride length condition (*p* < 0.01; *η*^2^ = 0.36). *Post-hoc* analysis revealed that the ball velocity of the NS was significantly greater than the US (*p* = 0.03; *d* = 0.78) and OS (*p* = 0.03; *d* = 0.79) but not significantly different between the US and OS (*p* = 1.00; *d* < 0.01).

**Figure 4:**
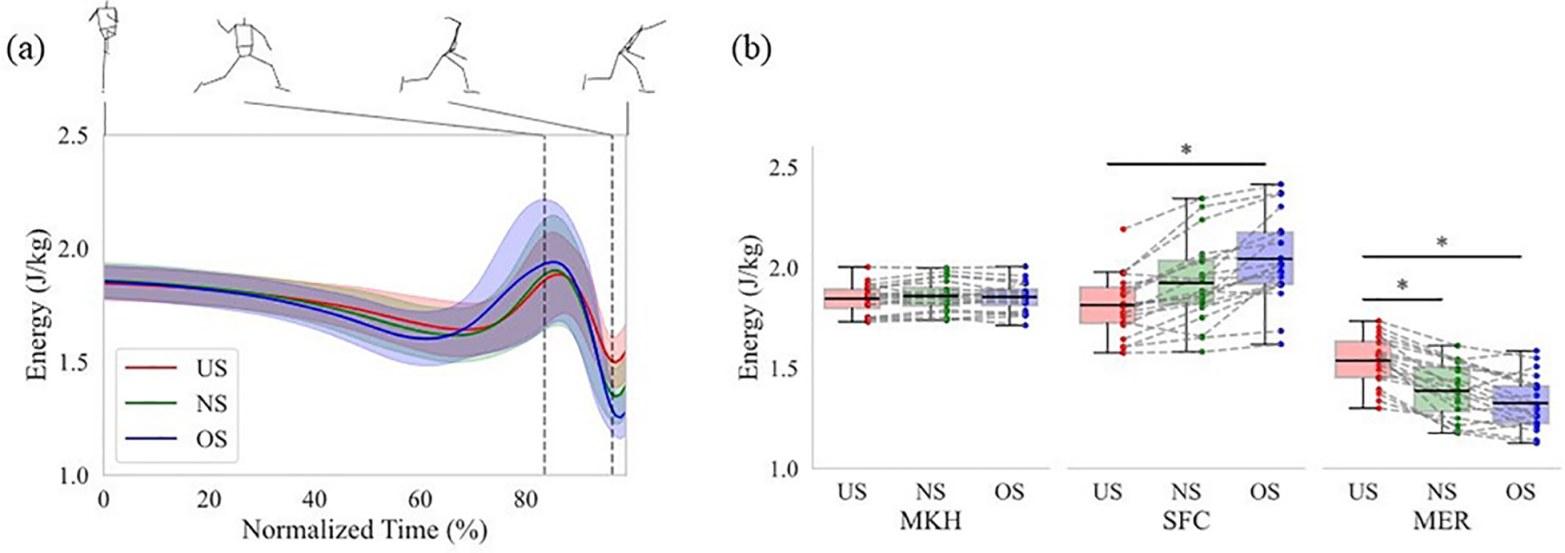
The mechanical energy of the lower torso. **(a)** Normalized time series data. The data from MKH to REL was normalized from 0% to 100%. The thick line shows the mean mechanical energy of the lower torso at each stride length. The thin line shows the statistical deviation of the mechanical energy of the lower torso at each stride length. The left and right dotted vertical line in the figure shows the time of the SFC and MER, respectively. **(b)** The data at each event. The box plot shows the interquartile range of the mechanical energy of the lower torso at each event. The black line in the box plot shows the mean mechanical energy of the lower torso at each event. The upper bar of the box plot shows the range from the third quartile to the maximum of the mechanical energy of the lower torso at each event, while the lower bar shows the range from the first quartile to the minimum. The dot plot shows the individual participant's mechanical energy at each event. The dotted line shows the same participant's mechanical energy with each stride length. The asterisk (*) shows a significant difference (*p* < 0.05) across the stride length.

The main effects were confirmed for both events or phases and stride length conditions in terms of the mechanical energy, the work of the *SP* of the lower torso, and the work of the *JP* at each joint. Therefore, this study examined the differences in each variable according to the stride length condition at each event or during each phase. Figure 4a shows time series data on the mechanical energy of the lower torso, and Figure 4b shows the mechanical energy of the lower torso at each event. There was no significant difference in mechanical energy of the lower torso between all stride lengths at the MKH. On the other hand, there were significant differences in mechanical energy of the lower torso between stride length at the SFC and MER. The mechanical energy of the lower torso at the SFC for the OS was significantly greater than that for the US (*p* < 0.01; *d* = 1.23). The mechanical energy of the lower torso at the MER for the US was significantly greater than that for the NS (*p* < 0.01; *d* = 1.65) and OS (*p* < 0.01; *d* = 1.00). The difference between the mechanical energy of the lower torso at SFC and MER increased with extending the stride length. In other words, the energy outflow from the lower torso increased between the SFC and MER. The energy flow during each phase was analyzed to reveal these results in detail.

**Figure 5:**
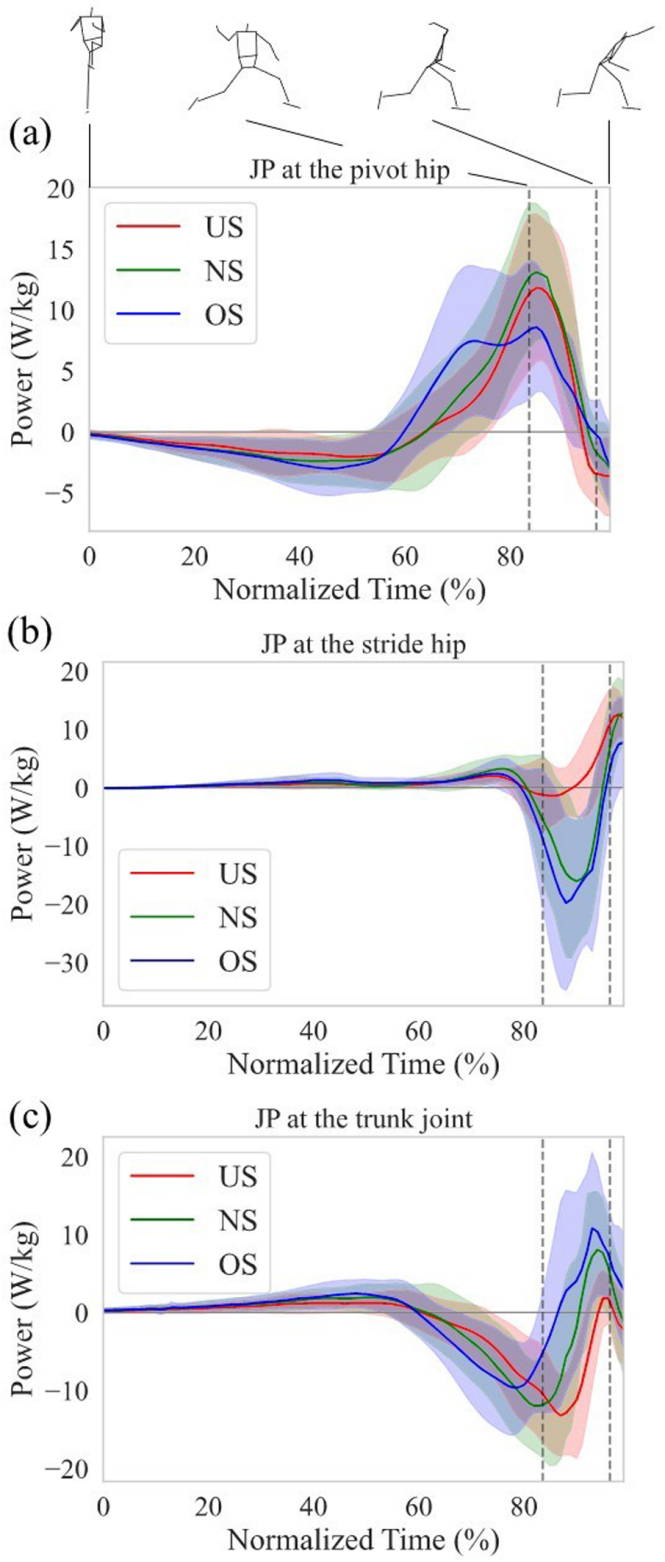
Normalized time series data of the *JP* at **(a)** the pivot hip, **(b)** the stride hip, and **(c)** the trunk joint. Each data from the MKH to the REL was normalized from 0% to 100%. The thick line shows the mean *JP* at each stride length. The thin line shows the SD of the *JP* at each stride length. The left and right dotted vertical line in the figure shows the time of the SFC and MER, respectively.

**Figure 6:**
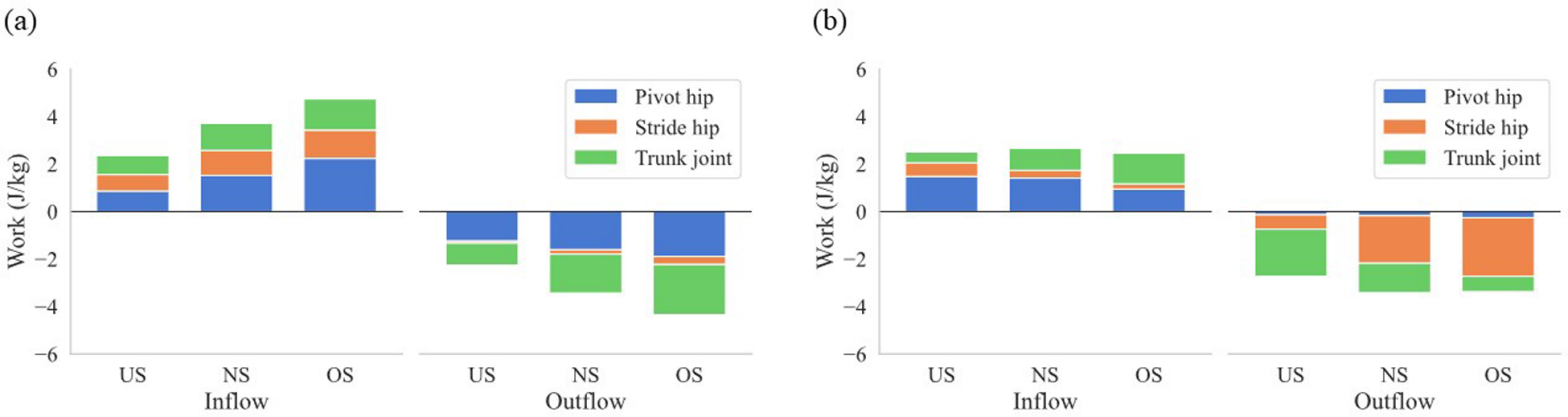
The mean work of the *SP* of the lower torso and *JP* at the pivot hip, the stride hip, and the trunk joint. **(a)** Shows the stride phase, and **(b)** shows the arm-cocking phase. Inflow shows the energy inflow to the lower torso, and Outflow shows the energy outflow from the lower torso during each phase. The total bar shows the mean work of the *SP* of the lower torso, and the amount of each color bar shows the mean work of each *JP* with stride length.

Figure 5a–c shows the *JP* at the pivot hip, the stride hip, and the trunk joint, respectively. The *JP* at the pivot hip increased earlier with extending stride length (Figure 5a), while the *JP* at the trunk joint decreased earlier with extending stride length (Figure 5c). To further elucidate how the energy flow differed between stride length conditions, the work of the *SP* and *JP* of each joint was analyzed. Figure 6a,b show the mean work of the *JP* at each joint with stride length during the stride phase and arm-cocking phase, respectively, and the amount of the total bar shows the mean work of the *SP* with stride length. The positive work of the *SP* increased with extending stride length, and the proportion of the pivot hip was greater than that of the stride hip and trunk joint, regardless of stride length (Figure 6a). The positive work of the *JP* at the pivot hip was greater for the OS compared to the US (*p* < 0.01; *d* = 2.18) and NS (*p* < 0.01; *d* = 1.01), and it was also greater for the NS compared to the US (*p* < 0.01; *d* = 1.03). The negative work of the *SP* increased with extending stride length. However, the proportion of the pivot hip was greater than the other two for the US in the negative work of the *SP*, while the proportion of the trunk joint was greater than the other two for the OS. The negative work of the *JP* at the pivot hip was greater for the OS compared to the US (*p* < 0.01; *d* = −1.93) and NS (*p* = 0.02; *d* = −0.94), and it was also greater for the NS compared to the US (*p* < 0.01; *d* = −1.15). The negative work of the *JP* at the trunk joint was greater for the OS compared to the US (*p* < 0.0; *d* = −2.11) and NS (*p* = 0.03; *d* = −0.74), and it was also greater for the NS compared to the US (*p* < 0.01; *d* = −1.21).

During the arm-cocking phase, the *JP* at the stride hip showed large negative values for both the NS and OS, while the negative values were small for the US and transitioned to positive values earlier (Figure 5b). The negative work of the *JP* at the stride hip was significantly lower for the US compared to the NS (*p* < 0.01; *d* = 1.86) and OS (*p* < 0.01; *d* = 2.66). In contrast, the positive work of the *JP* at the stride hip was significantly greater for the US compared to the NS (*p* < 0.01; *d* = 1.31) and OS (*p* < 0.01; *d* = 1.77). The energy outflow at the stride hip decreased with the shortening stride length, while the energy inflow at the stride hip increased with the shortening stride length (Figure 6b). The *JP* at the trunk joint during the arm-cocking phase transitioned to positive values earlier and increased with longer stride length (Figure 5c). The negative work of the *JP* at the trunk joint was significantly greater for the US compared to the NS (*p* < 0.01; *d* = −1.29) and OS (*p* < 0.01; *d* = −2.15), and it was greater for the NS compared to the OS (*p* = 0.01; *d* = −0.85). In contrast, the positive work of the *JP* at the trunk joint was significantly greater for the OS compared to the US (*p* < 0.01; *d* = 2.85) and NS (*p* = −0.90), and it was greater for the NS compared to the US (*p* < 0.01; *d* = −1.11). The energy outflow at the trunk joint decreased with extending stride length, while the energy inflow at the trunk joint increased with extending stride length (Figure 6b).

## 4 Discussion

This study investigated how altering stride length changes the energy flow from the lower extremity to the trunk. We found that the energy inflow from the pivot hip to the lower torso and outflow from the lower torso to the stride hip changed with stride length. However, the total energy outflow from the lower torso to the trunk joint during both phases was not significantly different across stride length conditions. These findings suggest that altering stride length changes the energy flow from the lower extremity to the lower torso without changing the total energy outflow from the lower torso to the trunk joint.

At first, this study defined the normal stride length. The mean stride length in the NS was 78.20% body height, which was consistent with previous literature reporting a range of stride length from 70% to 88% body height (12, 29–32). Furthermore, the mean ball velocity in the NS was 33.90 m/s, which was consistent with previous studies focusing on mature baseball pitchers (2, 33, 34). These results indicate that this study successfully recruited mature baseball pitchers.

The mechanical energy of the lower torso in pitching was altered with stride length condition (Figure 4a). Although the mechanical energy at the MKH was not significantly different across stride length conditions, the mechanical energy at the SFC for the OS was significantly greater than that for the US (Figure 4b). This indicates the difference between the mechanical energy at the MKH and SFC was greater for the OS compared to the US. In other words, the mechanical energy retained in the lower torso during the stride phase increased with extending the stride length. The mechanical energy decreased at MER, regardless of the stride length condition. The difference between the mechanical energy at the SFC and MER increased with extending the stride length (Figure 4b). This suggests that the mechanical energy outflow exceeds the mechanical energy inflow during the arm-coking phase as opposed to the stride phase. To clarify these results in detail, the energy flow was analyzed.

### 4.1 Energy flow during the stride phase

Our findings show that the increased energy inflow from the pivot hip to the lower torso with extending the stride length led to a greater total energy inflow to the lower torso during the stride phase (Figure 6a). It is assumed that the pivot leg acts as the driver of the pitching motion, as it pushes the body toward the home plate (35, 36). Therefore, the pivot leg needs to push more to get the over-stride length. It has been reported that the peak propulsive GRF by the pivot leg increased with extending the stride length (13). This force was correlated with the energy inflow from the pivot hip to the lower torso (36). Extending the stride length produces a greater propulsive GRF, pushing the body toward the home plate and increasing energy inflow from the pivot hip to the lower torso, increasing the total energy inflow to the lower torso during the stride phase.

However, extending the stride length increased total energy outflow from the lower torso due to earlier energy outflow to the trunk joint during the stride phase. Our results showed that the total energy outflow from the lower torso also increased with extending the stride length during the stride phase (Figure 6a). Specifically, the energy outflow from the lower torso to the trunk joint increased with extending the stride length (Figure 6a). Focusing on energy outflow from the lower torso to the trunk joint at the power level, the *JP* at the trunk joint became negative earlier (Figure 5c). This suggests that extending the stride length did not increase the magnitude of negative *JP*, but the earlier *JP* transition to negative and the prolonged duration of negative *JP* contributed to the increase in energy outflow from the lower torso to the trunk joint. The *JP* consists of *JFP* and *STP*, each derived from kinematic variables such as velocity and angular velocity and kinetic variables such as force and torque. Therefore, extending the stride length may have caused the kinematics and kinetics at the trunk joint to occur earlier. It has been reported that extending stride length led to a greater rotational angle of the pelvis and upper torso at the SFC, indicating early rotation of the pelvis and upper torso during the stride phase (37). To support this finding, examining the details of kinematics and kinetics timing in pitching when altering stride length is necessary.

### 4.2 Energy flow during the arm-cocking phase

**Figure 7:**
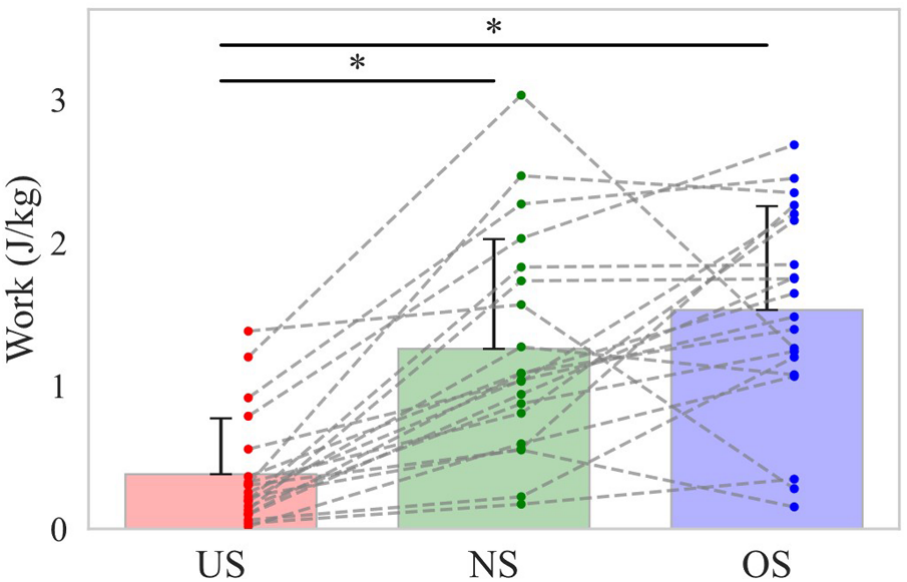
The energy absorption at the stride hip during the arm-cocking phase. The bar shows the mean energy absorption at the stride hip during the arm-cocking phase with each stride length. The error bar shows the SD of energy absorption with each stride length. The dot plot shows the individual participant's energy absorption with each stride length. The dotted line shows the same participant's energy absorption with each stride length. The asterisk (*) shows a significant difference (*p* < 0.05) across the stride length.

Shortening stride length reduced the increase in total body linear momentum during the stride phase, reducing the braking force by the stride leg and the energy absorbed at the stride hip during the arm-cocking phase. Our findings show that the energy outflow from the lower torso to the stride hip decreased with shortening stride length during the arm-cocking phase (Figure 6b). The body's COM accelerates toward the home plate during the stride phase, rapidly decreasing to rotate the trunk at SFC. During the arm-cocking phase, the pitcher absorbs the mechanical energy at the stride hip to reduce the COM velocity and create a stable base to rotate the trunk (20). Looking at the relationship between these factors and stride length, it has been reported that shortening stride length reduced the increase in total body linear momentum and the breaking force by the stride length compared to extending the stride length (13). Therefore, it seems that shortening the stride length decreased the total body linear momentum during the stride phase compared to other stride lengths, reducing the braking force by the stride leg and the energy absorbed at the stride hip to decrease the COM velocity during the arm-cocking phase. In this study, additional analysis of energy absorption at the stride hip during the arm-cocking phase revealed that it was lower in the US compared to the NS and OS conditions (Figure 7).

On the other hand, shortening the stride length increased the energy outflow from the lower torso to the trunk joint during the arm-cocking phase without increasing the energy outflow from the lower torso to the stride hip. As seen in Figure 5c, the *JP* at the trunk joint transitioned from negative to positive later with the shortening stride length during the arm-cocking phase. Energy outflow is represented as the integral of negative *JP*. This means that energy outflow increases by increasing the magnitude of negative *JP* and extending its duration. Therefore, the increase in the energy outflow from the lower torso to the trunk joint with the shortening stride length during the arm-cocking phase is likely due to the delayed transition from negative to positive *JP*, indicating a longer duration of negative *JP*.

### 4.3 The relationship between stride length and ball velocity

**Figure 8:**
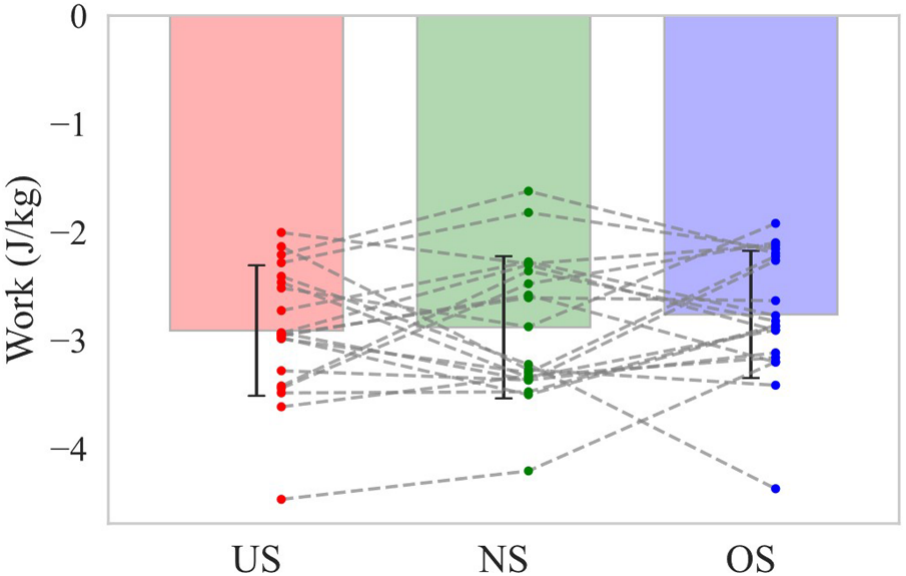
The total energy outflow from the lower torso to the trunk joint during the stride and arm-cocking phase. The bar shows the mean total energy outflow from the lower torso to the trunk joint during the stride and arm-cocking phase with each stride length. The error bar shows the SD of total energy outflow with each stride length. The dot plot shows the individual participant's total energy outflow with each stride length. The dotted line shows the same participant's total energy outflow with each stride length.

Baseball pitching is recognized as a kinetic chain in which the mechanical linkages of body segments allow for the sequential transfer of forces and energy from the lower extremity to the hand that ultimately releases the ball (14–16). You might think that if the energy inflow from the pivot leg to the lower torso, which is the primary inflow source, increases the energy outflow from the lower torso to the trunk joint would also increase. As shown above, the energy outflow from the lower torso to the trunk joint increased with extending stride length during the stride phase, whereas it increased with shortening stride length during the arm-cocking phase (Figure 6a,b). It is possible that the change in stride length altered the timing of energy outflow from the lower torso to the trunk joint. Therefore, the total energy outflow from the lower torso to the trunk joint during the stride phase and the arm-cocking phase was additionally analyzed and found to be not significantly different across stride length conditions (Figure 8). This suggests that although altering stride length changes the energy inflow from the lower extremity to the lower torso, it might not change energy outflow from the lower torso to the trunk joint.

It is difficult to predict ball velocity based on the lower extremities and torso results, which do not directly interact with the ball. Our results showed that altering stride length changes the energy inflow from the lower extremity to the lower torso. The ball velocity would be expected to correspond with the energy inflow from the pivot leg to the lower torso if this variable directly influenced ball velocity. However, the ball velocity was not significantly different between the US and OS, while the NS was higher than the other conditions in this study. A lot of studies have reported that increasing the kinematics, kinetics, and energetics of the lower extremity contributes to generating higher ball velocity in pitching (4, 5, 7, 19, 22, 32). The findings of these previous studies do not correspond to our results. Many segments exist between the lower extremities and the throwing hand that ultimately release the ball. Then, the ball velocity would not increase unless fully transferred to the hand, even if the lower extremity generates more energy. As the total energy outflow from the lower torso to the trunk joint did not vary across stride length conditions in this study, it seems that increasing ball velocity requires not only increasing the energy generated by the lower extremity but also efficiently transferring to more distal segments without loss. Further research focusing on the energy flow of the trunk and upper limb is hoped to clarify the relationship between each energy flow and ball velocity.

### 4.4 Limitations

This study had some limitations. First, it might not be generalized to youth because they might not have solidified their form. If the form has not been solidified, even the same individual would likely show significant variations in their movements across trials, making it uncertain whether consistent results can be obtained in repeated measurements. To address this issue, it would be valuable to collect data from participants with a wide range of performance levels and ages in future studies. Second, this study was conducted on a force-flat form in an indoor laboratory. Previous studies noted the difference in kinematics and kinetics in pitching from a mound vs. flat ground (38, 39). Therefore, the results of this study might not be generalized to the pitching from a mound. Third, this study was conducted to pitch to the target, not the catcher, because it is difficult to tell whether the ball reached the target zone depending on where and how the catcher catches it. It cannot be denied that the results of this study could be generalized to pitching to a catcher, as in a real game.

## 5 Conclusion

We found that extending the stride length increased the energy inflow from the pivot hip to the lower torso and the energy outflow from the lower torso to the trunk joint during the stride phase. This suggests that much energy was already outflowing from the lower torso to the trunk joint during the stride phase, even though a significant amount of energy was inflowed from the pivot leg to the lower torso. Additionally, we observed that shortening stride length decreased the energy outflow from the lower torso to the stride hip, whereas increasing the energy outflow from the lower torso to the trunk joint during the arm-cocking phase. The total energy outflow from the lower torso to the trunk joint did not significantly vary with stride length, and the ball velocity did not significantly differ between extending and shortening stride length. This study highlights that altering stride length might not lead to changes in total energy outflow from the lower torso to the trunk joint, implying difficulties in explaining ball velocity only by the lower extremity mechanics.

## Data Availability

The raw data supporting the conclusions of this article will be made available by the authors, without undue reservation.
